# Physical Literacy - A Journey of Individual Enrichment: An Ecological Dynamics Rationale for Enhancing Performance and Physical Activity in All

**DOI:** 10.3389/fpsyg.2020.01904

**Published:** 2020-07-28

**Authors:** James R. Rudd, Caterina Pesce, Ben William Strafford, Keith Davids

**Affiliations:** ^1^Research Institute for Sport and Exercise Sciences, Liverpool John Moores University, Liverpool, United Kingdom; ^2^Institute for Health and Sport (IHES), Footscray Park Campus, Victoria University, Melbourne, VIC, Australia; ^3^Department of Movement, Human and Health Sciences, University of Rome “Foro Italico”, Rome, Italy; ^4^Sport and Human Performance Research Group, Faculty of Health and Wellbeing, Sheffield Hallam University, Sheffield, United Kingdom

**Keywords:** non-linear pedagogy, athletics skills model, motor learning, ecological psychology, executive function, physical education, sport coaching

## Abstract

Internationally, governments, health and exercise practitioners are struggling with the threat posed by physical inactivity leading to worsening outcomes in health and life expectancy and the associated high economic costs. To meet this challenge it is important to enhance the quality, and quantity, of participation in sports and physical activity throughout the life course to sustain healthy and active lifestyles. This paper supports the need to develop a physically literate population, who meaningfully engage in play and physical activity through the development of functional movement skills in enriched environments. This is a shift away from reductionist approaches to physical activity engagement and maintenance to an ecological dynamics approach that focuses on enrichment to support functional movement skill learning and development. This is an embedded approach to physical literacy that allows learners the space and time to “explore–discover” (ecological psychology) within environments that will lead to a concomitant self-organization of highly intricate network of co-dependent sub-systems (anatomical, respiratory, circulatory, nervous, and perceptual-cognitive) resulting in functional movement solutions for the performance task and enduring positive adaptations to subsystems supporting the physical literacy journey across the life course. “Explore-discover adapt” is at the heart of two contemporary learner-centered pedagogies: Non-linear Pedagogy (NLP) and the Athletic Skills Model (ASM). Both emphasize the importance of enrichment experiences from an early age, and throughout life course, and both appreciate the inherent complexity involved in the learning process and the importance of designing a rich and varied range of athletic, participatory experiences that will support the embedded development of physical literacy leading to ongoing physical activity for all. The final part of this paper will demonstrate the potential of an ecological dynamics approach for supporting the concept of physical literacy by providing a roadmap for a reliable and valid measurement of physical literacy when considered from both an ecological dynamics perspective and the phenomenology understanding of physical literacy.

## Introduction

Across the globe more than 1.4 billion adults do not meet the recommended levels of physical activity (Guthold et al., 2018). The international pandemic of people leading sedentary lives is responsible for over five million deaths and an economic burden in excess of £50 billion per year (Lee et al., 2012; Ding et al., 2016). According to Ward et al. (2019) by 2030 in the United States of America: (i) one in two adults will be obese; (ii) the prevalence of obesity will be higher than 50% in 29 states and not below 35% in any state; and (iii), nearly one in four adults is projected to have severe obesity. Across the world health and exercise practitioners and academics, such as physical educators and exercise scientists, are challenged to resolve the massive threat posed by physical inactivity levels to the quality of people’s lives. Given the impact of this global health pandemic on life expectancies and quality of life, as well as the internationally burgeoning health bill, some have advocated a shift away from individualized approaches to behavior change and small scale individual quick fix interventions, to large scale systems-based interventions that are outward facing, prioritizing the role that the design of environments plays in sustainable physical activity promotion (Leone and Pesce, 2017; Ding et al., 2020). Referring to Baumann’s “liquid society,” Abu-Omar et al. (2019) have suggested that contemporary physical activity policies have entered a “liquid” age, in which promotion efforts are global and multi-sectoral. They have called for a balance of this all encompassing view with more focused approaches which should have a more limited and identifiable scope and, therefore, potentially greater effectiveness.

**FIGURE 1:**
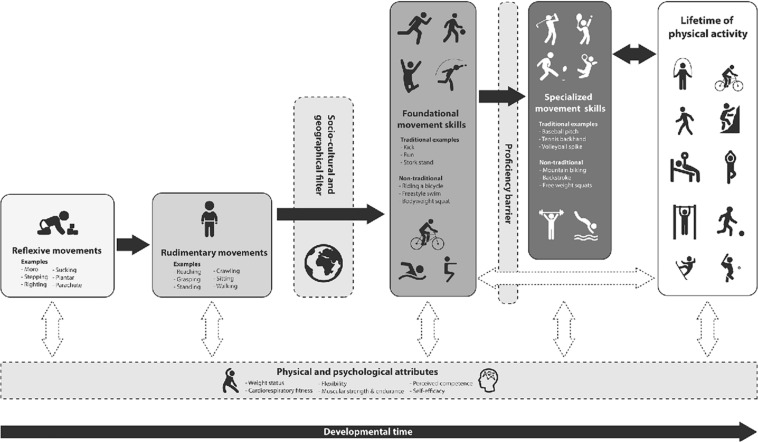
Development of foundational movement skills supporting physical activity across the lifespan (Hulteen et al., 2018).

In line with suggestions of Davids et al. (2016) it is important to enhance the quality, as well as the quantity, of participation in sports and physical activity throughout the life course. A well-defined and important scope for applied scientists and practitioners, exercise scientists and physical educators is to supporting children and adults of all ages to develop and maintain meaningful engagement in play and physical activity through the development of functional movement skills (Figure 1). Being competent in a broad range of movement skills is essential to promote enjoyment of, and engagement in, a range of different sports, physical activity and exercises in order to sustain healthy and active lifestyles across the lifespan (Robinson et al., 2015). Thus, the learning of movement skills which can enhance children’s functionality (i.e., capacity to participate meaningfully) in play, games and activities can: (i) contribute to positive trajectories of physical, mental and socio-emotional health throughout childhood (Robinson et al., 2015) and (ii) underpin the acquisition of skills and expertise needed by athletes to perform at a high level in sports (Savelsbergh and Wormhoudt, 2019) (iii) support all individuals at any stage of the life course in participation in sport, physical activity and exercise at a recreational level (Hulteen et al., 2018).

## Movement Skills Embedded: an Ecological Dynamics Rationale to Support the Emergence of Physical Literacy Capacities Across the Life Course

Children and adults who have acquired a broad range of movement skills are easily recognizable by the way they read and interact with their environment, for example moving to avoid an object, jumping over a puddle or kerb in the street or intercepting a ball at full stretch in the playground or on the sports field (Whitehead, 2001). The physical literacy journey is an enduring and constant process that begins at the start of life when we are unable to travel from one place to another, or coordinate our limbs to feed ourselves. Over the next decade, given opportunity to meaningfully invest in physical activity, children will develop, expand, and refine their physical, perceptual and cognitive capacities through performing functional movement skills during play making the transition from merely surviving, to thriving through meaningful embodied engagement in their environment and context (Adolph and Hoch, 2019). However, in many countries a high proportion of children do not have sufficient opportunities to engage meaningfully in physical activity and physical activity progressively declines while sedentary behavior increases, typically from the age of school entry (Van Hecke et al., 2016; Colley et al., 2017; Pearson et al., 2017). The on-cost of this problem is that today, children do not have as functional a movement skills repertoire as possessed their parents, or grandparents, and with it other integral aspects of physical literacy fall by the way side (physical, perceptual, and cognitive capacities) which previous generations acquired subconsciously through play. In summary, today’s generation of children are unlikely to engage as meaningfully in lifelong physical activity as earlier generations (Barnett et al., 2011; Tester et al., 2014; Bardid et al., 2015). Empirical evidence also suggests that children who do have well-developed functional movement skills seek out physical activity and experience better health outcomes compared to their peers who present low physical activity and high sedentary behavior levels (Robinson et al., 2015).

Ecological dynamics has been proposed as a framework to understand how we learn and acquire functional movement skills and how to create and structure enriched environments to support and cultivate a lifelong engagement in physical activity (Button et al., 2020). This perspective, espouses an embedded role for physical, cognition, emotions, and perceptual skills in the motor learning process. Ecological dynamics emerged from the work of Davids et al. (1994), Araújo et al. (2006), and Warren (2006) which highlighted multidisciplinary intersections between ecological psychology and dynamical systems theory. Ecological psychology suggests that learning movement skills is not predicated on processing information and accruing symbolic representations such as a movement template or schema, but rather on the continuous perceptual regulation of the learner’s action in a learning context. From a dynamical systems perspective, functional movement solutions emerge from the interactions of multiple sub-systems within the person, task and environment (Thelen, 1989; Davids et al., 2008). All sub-systems spontaneously self-organize, or come together and interact in a specific way to discover and explore an efficient, effective, and functional movement solution for each specific task (Thelen, 1989; Davids et al., 2008). Children learn to perceive affordances at each moment, relative to their current intrinsic dynamics (including skill competence and cognitive development) in the current performance environment and for the current task (Adolph and Hoch, 2019). Furthermore, system changes involve a non-linear process, meaning that children do not acquire functional movement skills at a steady rate (Chow et al., 2016). A small, but critical change in one sub-system will cause a cascade across the whole system, resulting in the emergence of a new movement solution during exploratory activity (Davids et al., 2003; Chow et al., 2016). As a function of learning and experience, movement skills tend to stabilize in an attractor state within the dynamic system. In complex dynamic systems, learning results in synergy formation between system components, such as muscles, joints and limb segments, and synaptic connections in the brain, resulting in adaptations across the whole system to support a child, or adult, to thrive in their environment and seek out opportunities to be physically active (Chow et al., 2011).

In summary, learning involves constraints-induced synergy formation of physical literacy capacities (physical, cognitive, emotional, and perceptual) through exploration, invention and adaptation of action possibilities. For scientists, academics and practitioners observing children in their natural settings, it is important to understand how different types of constraints (related to the task, individual and environment) converge to enable synergy formation for utilization of affordances (i.e., opportunities or invitations for action in the environment; Pinder et al., 2011). Rich informal play environments is therefore essential for children’s learning and development leading to a physically literate life. The next section will investigate how we can develop pedagogies and programs to maximize physical literacy for all.

## Meaningful Engagement in Physical Activity Through the Life Course: Ecological Dynamics and Environmental Enrichment

A key to sustainable engagement in physical activity is “enrichment”: the design of a rich and varied range of athletic, participatory experiences, opportunities, challenges, and activities that will require skill adaptation (Button et al., 2020) across a practice landscape from generality – specificity (Woods C. T. et al., 2020). In the next sections, we suggest how enrichment in physical activity has been operationalized through an ecological dynamics rationale and has led to innovation of two contemporary learner-centered pedagogies, that employ non-linear principles of learning: Non-linear Pedagogy (NLP) and Athletic Skills Model (ASM), each of which reflects the inherent complexity involved in the learning process. Both pedagogical approaches emphasize the importance of enrichment experiences from an early age, and throughout the lifespan, to facilitate a generality, and later specificity of skill adaptation needed to engage in and maintain involvement in high performance sports through to recreational physical activities (Seifert et al., 2019; Stone et al., 2018; Strafford et al., 2018). As was the case for the learning of functional movement skills, “Enrichment” can be understood as an embedded approach to motor development and learning, emphasizing the importance of rich and varied possibilities to achieve task goals through interactions constrained by the body, task and environment (Renshaw and Chow, 2019). The learning interactions created through enriched environments lead to quality physical activity that will support adaptations to cognitive functions that, in time, have shown signs of transfer to support children’s in academic achievement (Schmidt et al., 2017). To this aim, we shift the focus from “cool” executive functions, which are elicited under decontextualized and emotionally neutral conditions and studied from a cognitive perspective to “hot” executive functions, which are enacted in emotionally salient contexts and studied from a broader socio-emotional perspective of cognition (Zelazo et al., 2010). Such an understanding of cognition accounts for the non-linearity in cognitive development and fits with the individual’s embodied/embedded physical literacy journey (Whitehead, 2001; Rudd et al., unpublished). This is encapsulated by Kelso et al. (2013) “*everyone’s neural network is different. The nodes are different, the connections too, individual differences exist at all levels of structure and function. This means that children (or any human) will subtly walk differently, think differently and feel differently and critically respond to the environment differently.*” Therefore to support physical literacy across the lifespan where play opportunities have diminished we need pedagogies that account for such variability and individual difference in the learning process.

### Non-linear Pedagogy

Ecological Dynamics have led to the creation of learner-centered pedagogical principles which cater for individual needs and emphasize an “explore–discover–adapt” learning approach called “NLP” (Chow et al., 2016). The predicted long-term effect of this pedagogical approach is that children will acquire a wide range of functional movement solutions that are both adaptable and attuned across physical activity environments (Chow and Atencio, 2014). Learning and practice are considered as a search for synergies such as functional performance solutions which are uniquely adapted to individual learners. Learners search to utilize “affordances” offered by the environment (Gibson, 1979). Affordances are opportunities or invitations for actions in the form of functional performance behaviors which can achieve specific intentions and goals, examples in sports and physical activities might include climbing a vertical surface (e.g., where affordances for support and traverse are offered by gaps, ledges and cracks in the surface of a tree) or intercepting a moving object (e.g., where the trajectory of a moving object invites an interception with one or more limbs, like the fingers, hands, arms, or legs). Continued experiences of interactions with objects, events, and significant others in a performance environment provide opportunities for learners to perceive and utilize new affordances, leading to adaptations of developing skills, enhancing performance functionality. Practice task designs for learners in sport and physical activity need to be coherent, innovative and challenging in order to *invite* specific patterns of behavior through the coordinated activity of learners. The aim of structured activity programs is to: (i) provide experiences and opportunities for individuals to enrich sub-systems which can help them perceive and utilize a broad range of affordances of a performance environment; and, (ii), engage in specialized learning experiences which can help them enhance their expertise in specific activities (Rudd et al., 2020).

#### Conceptual Model and Principles of Non-linear Pedagogy

Non-linear Pedagogy outlines five principles for the design of such practice tasks: *representativeness, constraints manipulation, task simplification, informational constraints, and functional variability* see Rudd et al. (2020).

The design of *representative* learning environments for individual learners requires a deep understanding of the information that regulates performance behaviors and the affordances that may invite specific functional actions needed to achieve intended task goals. Learning designs can be made more effective by including relevant information and affordances should emerge during the learning process. While *constraints manipulation* is critical in providing boundaries which afford specific movement possibilities for learners to exploit existing movement tendencies, there should be a clear focus on *task simplification* to enable learners to develop and maintain strong functional couplings between information and movement during learning. In practice, information is directly perceivable and is able to be picked-up by individual learners to constrain actions (Davids et al., 2008). Skillful perception is, acquired by a process of searching for “specifying” information used to regulate movements (Gibson, 1979). Learners should be allowed to *interact* with a performance environment (constantly make decisions and move) to generate more information that can be used for regulating subsequent performance. In this way, learners can be sensitized to the impact that key *informational constraints* have on their emerging movement patterns. Practice variability plays a relevant role in challenging learners to explore different movement solutions and adapt their actions to the dynamics of information flows surrounding them in performance contexts (*functional variability*). A key challenge is to consider how to manipulate the amount of variability (in individual, task or environmental constraints) within, and between, learning sessions to challenge learners and enhance their self-regulatory capacities, rather than over-reliance on verbal directions from the coach or teacher (Chow et al., 2016). Below, we consider the effects of different directions of performance regulation on learners: known as global to local (coach-led) and local to global (learner-led) directions (Ribeiro et al., 2019). These design principles can operate through the key pedagogical channels of informational and practice constraints, with less attention being paid to verbal instructions to allow functional goal-directed behaviors to emerge in individuals.

**FIGURE 2:**
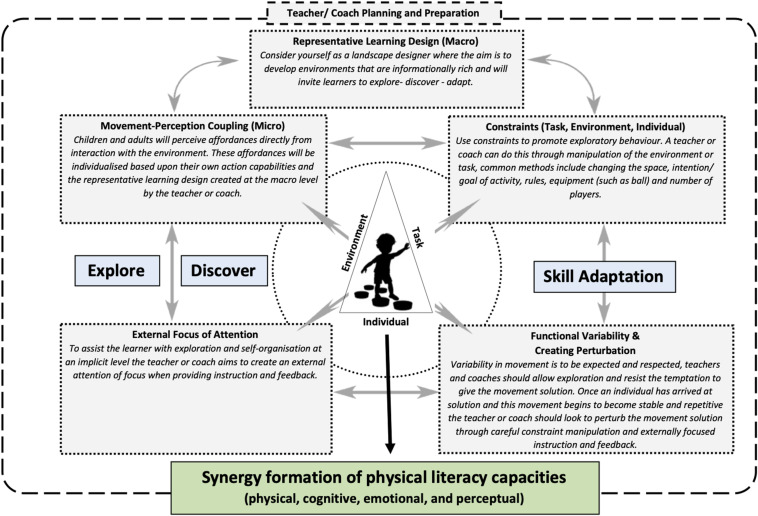
Non-linear pedagogy principles and how they can support physical literacy.

A meta-analysis by Cerasoli et al. (2014) reported positive relationships between enjoyment and performance due to populations being intrinsically motivated to persist at a task. It seems that being intrinsically motivated in a physical activity is crucial for sustained participation and that the obsession with frequency-based movement metrics, such as time, amount (e.g., counting steps), and distance provides a limited view of experiences. Being learner-centered, NLP should support the satisfaction of the basic needs of autonomy, relatedness and competence and therefore nurture the development of motivation (Moy et al., 2016; Lee et al., 2017). Indeed, it provides the child with choice and freedom which may enhance their enjoyment and perceptions of autonomy. The respect the teacher or coach gives to the child’s ability to explore, learn and problem solve may also enhance the child’s feelings of relatedness (Lee et al., 2017). The focus on finding different movement solutions to achieve a goal creates a task oriented climate and should, over time, generate a shift in how the child views competence, away from an “ideal” movement performance toward functional, creative movements (Moy et al., 2016; Lee et al., 2017). Indeed, employing NLP to assist in the design of physical activity environments will foster novel functional movement solutions and generate new opportunities for exploration that trigger developmental cascades across physical literacy capacities (Figure 2). It should be noted, however, that while NLP could support basic psychological needs, it would reject the notion that motivation is intrinsic. Instead it would view motivation as residing beyond the boundaries of the human body in that outward facing intentionality, as a perception of surrounding information, is direct and an external focus of attention is key to achieving the intended task goals, harnessing embedded, self-organized, highly interconnected, co-dependent, behavioral sub-systems.

#### How Skill Adaptation Is Promoted in Non-linear Pedagogy: Should We Use the Term Creativity in Movement Performance and Do Executive Functions Matter?

A neurocomputational, top-down, account of creativity proposes that creative actions are specifically emanate from creative thinking and ideas. Aligned with this view, measures obtained with tests of movement creativity have frequently been used as movement-based measures of creative *thinking*, rather than measures of domain-specific movement creativity (Scibinetti et al., 2011). One of the main assumptions in accounts of the neural underpinnings of creativity, is that tasks involving creative thinking induce changes in prefrontal cortical activity (Dietrich and Kanso, 2010). The rationale behind this perspective is that, since the prefrontal cortex is the main substrate of executive function, the relationship of creativity to core executive functions (inhibition, working memory, cognitive flexibility) is key. In particular, the prefrontal cortex is proposed to activate executive processes relevant to the ongoing task and to the goal-oriented expression of creative insights (Dietrich, 2004). The postulate, that executive control is needed prior to enacting a creative idea in the motor domain, has been challenged by recent evidence that the manipulation of working memory load does not affect the conduct of convergent and divergent tasks typically used to test motor creativity (Moraru et al., 2016; Orth et al., 2019). Processes like working memory, which is seemingly not crucial for a creative movement action to emerge, may exemplify “cool” executive functions that are assessed under decontextualized, emotionally neutral task conditions.

In recent years, an embodied/embedded view of cognition, grounded on sensorimotor coupling, and subserving action (Engel et al., 2013) and has provided the basis for a new perspective that brings creativity research closer to an ecological dynamics framework. Orth et al. (2017) proposed that creative actions are novel functional movement patterns that do not need the top-down, antecedent generation of a creative idea, but rather emerge in a bottom-up fashion from the exploration of adaptive solutions to motor problems.

Creativity, captured from an ecological dynamics rationale, is posited as a transformational process, involving, search, exploration and discovery of novel and functionally efficient behaviors for performance (Hristovski et al., 2009). In this view, movement output emerges from cooperating subsystems, exploiting self-assembly tendencies to form synergies at different levels, concomitantly self-organizing with other subsystems to achieve functional movement solutions (Raja and Anderson, 2019). This creative process in the context of coordination and its acquisition in athletes and sports teams is predicated on the continuous (re)formation of functional synergies between relevant system degrees of freedom during practice and performance (Woods C. et al., 2020). Ribeiro et al. (2019) highlighted how system degrees of freedom in athletes and sports teams are continuously (re)emerging in and out of synergies adapted to achieve movement goals and tactical behaviors. They proposed two main directions for synergy (re)formation in sport practice: global to local (synergy formation directed by an external agent such as coach or teacher) and local to global (when players self-organize under competitive performance constraints). These directional tendencies for self-organization of system degrees of freedom rely on a broad array of task and environmental constraints that must be perceived to effectively explore available opportunities for action.

During movement skill performance the inherent self-organization tendencies of subsystems is predicated on degeneracy and multistability (Edelman and Gally, 2001; Diamond, 2013). NLP appreciates the role of neurobiological properties of self-organization, degeneracy and multistability, ensuring that adaptive movement variability is an essential ingredient of physical activity, movement experiences and task practice to facilitate innovation in synergy formation (Hristovski et al., 2009; Chow et al., 2011). An observed output from the continuous adaptation of actions under changing constraints are movement innovations, emerging through interactions with the environment, task and individual constraints. In this ecological dynamics conceptualization, executive functions play an integral role in planning and preparation underpinning the intentionality, and “knowledge of” the environment (Woods C. T. et al., 2020) that frames the self-organization tendencies that emerge under interacting constraints. The deeply entwined relations of intentions, perception and action provides a boundary for the self-organization tendencies that can be exploited for the functionally innovative and adaptive actions during the continual transitioning between environmental information and the intrinsic dynamics in sport performance (Fajen et al., 2009; Hristovski et al., 2011; Araújo et al., 2019; Seifert et al., 2019).

How can these ecological dynamics concepts be exploited in competition and sport practice? Santos et al. (2016) proposed a game-centered, constraints-led approach as a means to promote the emergence of innovative and adaptive tactical behaviors in team sports practice. Further, during competitive performance the intentionality is to “*win or beat an opponent*” such a strong outward facing intentionality may be necessary for players to learn, within competitive situations will lead to perturbation and require self-organization by continuously adapting to dynamic performance constraints (Ribeiro et al., 2019). To support the emergence of local to global tendencies in competitive performance, researchers and practitioners could manipulate task and environmental constraints and evaluate the impact on aspects of physical literacy development as well as whole team synergies. Buszard et al. (2016) conducted a systematic review of equipment manipulations and play space in children’s sport and found that these adjustments to task constraints are advantageous to performing functional movement skills, compared to traditional global to local methods (teacher led). García-Angulo et al. (2020) found that manipulation of task constraints such as varying the pitch size, number of players or goal size enhanced football-specific self-efficacy. Buszard et al. (2017) found that an overemphasis on global to local instructions can be detrimental to children who have lower working memories, inhibiting their motor learning. This is, in turn, consistent with findings showing an advantage of global attention training for enhancing creative tactical solutions (Wyrick, 1968) and the special ability of highly skilled team players in using a global attentional focus (Pesce and Bösel, 2001) and performing local-to-global visual attention shifts (Pesce et al., 2016). The relevance of local to global practice designs in sport performance contexts suggests a novel intersection of key ideas in NLP with the less investigated subset of “hot” executive functions, the motivational salience of the context and emotional engagement as a distinguishing feature of “hot” executive functions for harnessing self-organization tendencies (Harms et al., 2014). NLP, applied to programs of work on team sports appropriately fosters “hot” decision making, underpinning a rapid synergy formation process amongst system components, and exploiting a “local to global” directional tendency within individual players and teams (Davids et al., 2013; Ribeiro et al., 2019) predicated on individual physical literacy. The key tenets of ecological dynamics, and its related principles of an NLP, are harmoniously aligned with ideas of a practitioner model of skills development and learning, such as the ASM, which we discuss next.

### Athletic Skills Model

**FIGURE 3:**
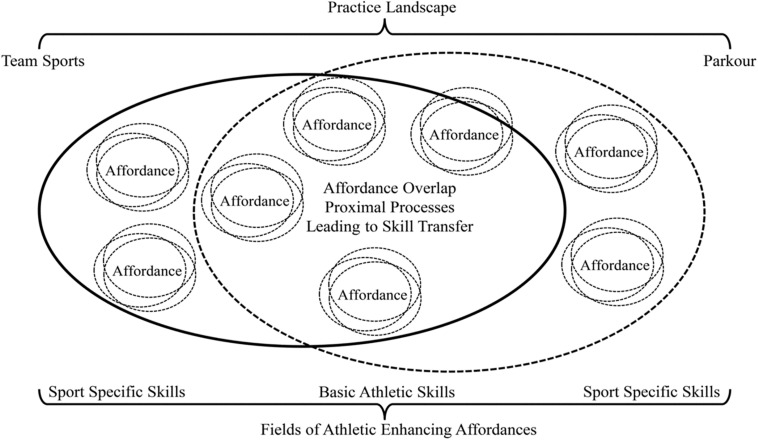
Overlap of performance-enhancing affordance fields between team sports and parkour as a donor sport (Strafford et al., 2018).

Traditionally organized physical activity, sport and physical education programs have been criticized for being overly structured, prescriptive and didactic in their organization and delivery (Kirk, 2010; Jess et al., 2017) which may cause negative perceptions and associations between children and physical activity and put children off physical literacy (Ntoumanis et al., 2004; Whitehead, 2010). This is problematic as participation in organized programs affects children’s engagement in on-going autonomous physical activity (Morgan et al., 2008). Such negative perceptions have also been noted with traditional pedagogical practice designs, emphasizing frequency-based metrics, such as number of hours spent in practice and implicated in high dropout rates recorded from development programs for talented youth athletes in high performance sports (Côté et al., 2011; Coutinho et al., 2016). The ASM is a pedagogical framework that offers an alternative program structure which emanated from professional sports practice, proposing how a diverse range of sport experiences can enrich later specialization in a sport (Wormhoudt et al., 2018). In this respect, it is an ideal contrast to models of early specialization, such as the deliberate practice approach. The ASM focuses on the development of functional movement skills and physical literacy capacities, advocating that early childhood play and practice experiences should involve participation in “multi-sports,” involving a diverse range of movement experiences and activities. In the “multi-sport” stage of the ASM, children need to experience and “sample” a broad variety of activities and sports which will enrich their athletic development, encouraging them to explore functional movement behaviors that enhance all their physical literacy capacities. [see Figure 3; (Kirk, 2005; Côté and Hancock, 2016)].

The holistic development of learners, when interacting with different fields of a performance landscape, contributes to their functional co-adaptation to changing environmental and task constraints (Araújo et al., 2010). A key aspect of learning design in the ASM is that activities should always involve pleasure and fun experienced through the constant exploration of different movement environments leading to novel individual action possibilities. Through play in different performance contexts, engagement in multiple sports and physical activities has the potential to increase the long-term motivation for practice, essential for later performance in elite sports, should learners want to continue to the highest performance levels (Wormhoudt et al., 2018). As mentioned earlier, an ecological dynamics rationale highlights that a major problem with early specialized, sport-specific training concerns the ubiquitous over-emphasis on specificity of transfer in practice at all levels, which is especially problematic for the physical and mental health of youth and children (Davids et al., 2017). The frameworks of ecological dynamics and the ASM provide a more nuanced understanding of transfer from training to performance in sport. It is suggested that the role of behavioral enrichment, conferred by *generality of transfer* has typically been misunderstood and undervalued in athlete development programs.

#### The Role of Multi-Sports and Donor Sports in Athlete Development

The ASM is a practitioner-led model of talent development and skill acquisition, proposing how, through “multi-sport” (rich, varied) experiences, movement competences can support transfer of five relevant performance components: agility, endurance, flexibility, power and stability. The ASM proposes that perception and action is organized into seven distinct abilities: adaptability, coupling ability, ability to react, rhythmic ability, balance ability, spatial orientation ability and the kinetic differential ability (Wormhoudt et al., 2018). These performance components and related abilities are, not only extremely relevant for developing skill and expertise in talented athletes, but also for recreational-level engagement, and maintenance of participation, in sport and physical activity throughout the lifecourse. The ASM clearly articulates that, early in learning, children should experience fun and enjoyment in *playing* a variety of physical activities and sport, which may not have a direct relationship to a target sport (Wormhoudt et al., 2018). Here, the central argument is that individuals become skillful by being exposed to a mix of non-specific and specific experiences, which support more functional capacities for later specialization in a specific target sport (Güllich, 2017). Integrating the key concepts of the ASM and the Ecological Dynamics approach to motor learning, thereby avoiding the consequences of early specialization, it is proposed that *Donor Sports* can enrich foundational skill development, relevant for later performance in a target sport (Savelsbergh and Wormhoudt, 2019). Opportunities to acquire relevant, athletic skills and abilities, such as balance, body awareness, coordination, reaction speed, strength and turning ability, can be “*donated*” through an affordance landscape shared between an athlete’s main target sport and donor sport activity (Strafford et al., 2018).

Sport practitioners should aim to integrate donor sports, into teaching and coaching programs, at all levels, to exploit the sharing of foundational movement skills required for an athlete to perform *well* in a target sport. Being exposed to donor sports early in learning could be particularly useful when particular skills required in a target sport, are considered to be under-developed in an individual athlete’s movement repertoire. Shared performance-enhancing affordances in the donor sport landscape present opportunities for the development of key athletic skills that may be instrumental for specialization in an identified target sport. For example, in team sports donor sports exposure may enhance skill transfer in the way that performance in both sports requires the dynamic (re)organization of body segments relative to the opposition, task goal [e.g., specific postural control and balance required for movement orientation and (re)orientation when challenging for the ball in team sports such as soccer and rugby]. This needs to be verified in future empirical research. Therefore, a challenging issue for researchers and practitioners concerns how to enhance the specificity of skill transfer through a donor sport which is representative of the functional coordination patterns shared with a target sport. Evidence suggests that donor sports may also be beneficial for the development of psychological skills, such as problem solving; stress relief; self-efficacy and risk management (see Strafford et al., 2020).

#### The Athletic Skills Model’s Emphasis on the Importance of Physical Activity Enrichment

Using donor sports to enrich the performance of learners in sport and physical activity provides the basis of how the ASM emphasizes the value of enrichment through experiencing a wide range of activities. In this section, we exemplify how the ASM’s advocacy of the role of donor sports has implications for how affordances landscapes in sports such as Parkour and Futsal can support skill development in team game players.

##### Parkour

Practitioners (known as Traceurs) are required to negotiate environmental properties and features of indoor or outdoor installations in the most innovative and efficient manner possible. Parkour’s two main distinctive disciplines are speed and free-style. Speed runs require Traceurs to move from a start to an end point in the quickest way possible, and free-style runs are judged on how creative the Traceurs are at moving around the installation. Parkour’s potential as a donor sport for athlete development has been recognized recently (Strafford et al., 2018, 2020). In team sports, practice designs encourage performers to effectively use dynamic and fluid movement patterns in representative game-based scenarios, with the dynamic (re)organization of body segments at different speeds, relative to movement of the ball and positioning of opponents in the surrounding landscape (Edwards et al., 2017; Seifert et al., 2019). Strafford et al. (2018) outlined how Parkour could provide an adaptive and affective platform for psychological, perceptual, physical and social development in team sport players. They proposed that Parkour style activities should be integrated into the athlete practice landscape to develop athleticism and skill transfer in youth team sports, such as soccer and rugby, through a shared network of affordances and foundational performance behaviors. A shared network of affordances in the environment may invite movement exploration, which in turn provides opportunities for the development of athletic skills relevant to the team sport game being targeted. In this sense, abilities that are deemed to be critical to athlete specialization in team sports can be “donated” by Parkour, such as creativity in negotiating gaps or obstacles, fluidity of movement and related decision-making or safe landing strategies.

According to the ASM, a coach facilitates the development of practice landscapes (Wormhoudt et al., 2018). Strafford et al. (2020) interviewed experienced Parkour Traceurs on the skills they believed were developed through parkour, also asking how they developed Parkour practice landscapes to support the development of physical, perceptual, psychological and social skills. Parkour Traceurs explained that, for athletic development, indoor parkour environments have to promote creative and exploratory movement behaviors, whilst physically and psychologically conditioning the athlete, through heightened opportunities for decision making and action functionality. Practically, Parkour Traceurs discussed how this is often achieved through the development of modular practice landscapes, where the spacing, orientation and angles of the installation blocks and bars set ups are manipulated to increase or decrease task difficulty.

The ideas outlined by Strafford et al. (2018, 2020) suggest that the development and design of playscapes require a fundamental and direct shift toward the inclusion of varied boundaries, limitations and features not typically found in modern playgrounds, which constrain the (re)organization of motor system degrees of freedom and different levels relative to each individual’s intrinsic dynamics (Bernstein, 1967; Sparrow and Newell, 1998; Sharma-Brymer et al., 2015). Parkour-style activities like “world-chase tag” could provide a platform for integrating Parkour playscapes into team sport settings^1^. For example, the global constraints governing world-chase tag (i.e., the first person to tag their opponent wins) are comparable to the offensive phases in Rugby Union, where to regain possession of the ball and score a try, athletes have to couple their movements relative to the positioning of their opponents, teammates and the direction of the ball.

##### Futsal as a donor sport for football

Futsal places a clear emphasis on controlling and manipulating the ball in a smaller relative space per player (RSP), compared to other team sports, like football and rugby. In futsal, performers require “soft feet” to manipulate the ball into tight spaces, using different segments of the foot, to dribble, pass and shoot the ball [which is smaller (size 3), and a lower coefficient of restitution than a regulation size 5 football ball; Araújo et al., 2004]. In this sense, futsal is distinct from football, with the latter’s emphasis on performance of gross motor skills and increased playing area dimensions (with associated differences in timing and coordination of actions). For this reason, footballers are required to have greater levels of relative strength and explosive power to move around larger pitch areas efficiently (with and without the ball), requiring coordination and dynamic (re)organization of body segments in relative space. With different playing area dimensions on a macro level (in terms of goal directed actions, goal scoring, covering space), futsal, on a micro level, promotes the development of skills required to perform *well* in some contexts of football, such as: balancing to react to sudden changes of direction on court, coordination for changes of direction and the capability to react quickly and rhythmically for the smoothing of perturbations. Further, similar to football, futsal requires performers to couple their body movement precisely with movements of teammates and the opposition, and the direction of the ball, through visual exploratory behaviors (known as “scanning” in team sports performance). Indeed, Oppici et al. (2017) observed that futsal players scanned for space and co-positioning of other players 54% of the time prior to taking the first touch, compared to 16% of the time in football players. Futsal also provides more frequent opportunities to perform skills and engage with the ball, compared to 11 a side football (Davids et al., 2013). In football and futsal, each player is required to use both feet to perform the skills needed during competitive performance, as well as to engage in collective tactical behaviors, with and without the ball (Travassos et al., 2018). Preliminary evidence suggests that the performance landscape shared between futsal and football implies that the former can act as a donor sport to enrich athletic skills relevant to the behavioral adaptability that footballers can exploit for skill transfer. Integrating futsal into football practice, could lead to enriched physical, perceptual and psychological skills, by emphasizing behavioral correspondence between the sports, and heightened behavioral adaptability developed by exploiting similarities in intentional constraints on performance (Seifert et al., 2019) although this needs to be substantiated in empirical research.

## An Emerging Discourse for Physically Active Futures: Physical Literacy and How It Aligns With an Ecological Dynamics Rationale

We began this paper by highlighting a concern with the current direction of physical activity research and the professionalization of children’s sport, and proposed an ecological dynamics rationale as an alternative discourse that focuses upon enrichment activities for each individual across the life course. These ideas lead us to consider physical literacy, a concept put forward by International Physical Literacy Association (2017) who defined physical literacy as “*the motivation, confidence, physical competence, knowledge and understanding to value and engage in physical activity for life*.” Over the last decade, the concept of physical literacy has gained traction in physical education, youth sport literature for the holistic development of physical, perceptual and cognitive skills to support long term engagement in physical activity (Sullivan et al., in press). Historically, physical literacy discourse emanated from physical education, in particular movement education literature, underpinned by the philosophy of phenomenology which contends that every individual has a unique embodied understanding of the world. This is embedded within their own experiences with the physical and mental being viewed as an indivisible, mutually enriching whole (Shearer et al., 2018). In recent times the concept of physical literacy has been found to be appealing to many, though it has been highlighted that the philosophical underpinnings are considered by many to be overly complex (Jurbala, 2015; Shearer et al., 2018). There are also claims that it is difficult to operationalize, leading to governments, policy makers, researchers and practitioners developing their own interpretations of physical literacy (Martins et al., 2020). Up to this point we have demonstrated that linking the ecological dynamics rationale and physical literacy can support long term engagement in physical activity through the integration and the adoption of NLP and ASM across physical education, sport and exercise context. The final section of this paper will demonstrate how ecological dynamics can provide a way to measure physical literacy which is one of the current most contentious issues facing the adoption of physical literacy.

A common misconception within physical literacy is the adoption of fundamental movement skills to capture children’s physical literacy (Rudd et al., 2019). Children perform skills in isolated and closed environments and are assessed on how well they conform to an optimal movement template (Ulrich, 2013; Rudd et al., 2016). This over-emphasis on technique is driven by the level of standardization required to make reliable comparisons across cohorts of children when using kinematic, process-oriented assessments. This data collection is vital as it is responsible for shining a light on the current poor levels of movement in children globally. However, when it is conceptualized from a physical literacy vantage point it is fraught with validity issues, from both a phenomenology philosophy perspective and from the ecological dynamics rationale (Araujo and Davids, 2009; Almond, 2014; Rudd et al., unpublished). An over-emphasis on technique diminishes perception and action of how children play in the game situation or in unstructured environments. This is critical, as in closed environments the self-organization of physical literacy capacities will be very different from the embodied experience of playing with friends.

Utilizing an ecological dynamics conceptualization of physical literacy, we can move toward a better, though still not perfect (in this paper anyway), solution to this conundrum, by utilizing an assessment which is both reliable and a valid measure of physical literacy. To develop a reliable movement assessment we require a standardized environment and task for all children; ecological dynamics requires the task to be outward facing in an open and informationally rich environment that is safe for exploration (Seifert et al., 2019; Hacques et al., 2020). There are already movement assessments being used that, whilst not developed from an ecological dynamics framework perspective, meet these requirements, for example the divergent movement assessment (Cleland, 1990; Rudd et al., 2020). Children are placed in a rich environment that has been set up based on a standardized protocols, in that every child is placed in the same environment and given two 90 s blocks to explore and interact with the environment in as many ways as they can. For each functional movement solution they demonstrate, they are rewarded with a score of one and to assist children with exploration, every 30 s children receive a predefined prompt from the research assistant. In summary, such a task does not prescribe a technical movement skill template but instead provides an inviting/enriched environment for children to interact and perform in. With a standardized environment and carefully considered task constraints it will be possible to gain an understanding of the invitations for action (affordances) which emerge that are functional for that child. Using this form of assessment a child in a wheelchair can demonstrate a far greater number of functional movement solutions based upon their own self organization constraints into functional movement solutions than a child who is performing on two feet but has had a poorer physical literacy journey to that point. This is because this assessment taps into the children’s exploration and displays how far they have come in terms of learning to learn how to move (Adolph and Hoch, 2019). This assessment is individualized and closer to the embodied experience in play (though again not perfect) and can be used to create established normative and group comparisons for a child’s physical literacy. This can support researchers in monitoring skills and practitioners in designing learning environments which support physical literacy using pedagodgies such as NLP and ASM.

## Conclusion

This paper provides applied scientists and practitioners with a new perspective on how to improve the quality, not just the quantity, of participation in sports and physical activity to enhance the health and well-being of future generations. It has proposed a shift away from reductionist approaches to suggest that humans can be conceptualized as deeply integrated complex systems. An individual’s, interaction with a task and their environment, through meaningful engagement in physical activity results in the performance of functional movement solutions and adaptations through the whole system (physical, cognitive, emotional, and perceptual). These adaptations across the whole system are advantageous in supporting children and adults on their journey to lead a physically literate life. Furthermore, an ecological dynamics rationale can provides a framework to support physical literacy program design using NLP (Chow, 2013) and the ASM (Wormhoudt et al., 2018) as well as highlighting the benefits of assessments such as the divergent movement assessment in measuring physical literacy compared to more controlled assessments such as the test of gross motor development (Cleland, 1990). Combining these enrichment experiences from an early age and throughout the lifespan, utilizing contemporary theory of skill acquisition, offers real hope to engage and maintain childrens’ and adults’ involvement in sports and physical activities across the lifecourse (Seifert et al., 2019; Stone et al., 2018; Strafford et al., 2018) with the ensuing health benefits.

## Author Contributions

JR and KD conceived the review. All authors were involved in the writing and revising of the manuscript and read and approved the final manuscript.

## Conflict of Interest

The authors declare that the research was conducted in the absence of any commercial or financial relationships that could be construed as a potential conflict of interest.

## Footnotes

1 https://www.youtube.com/watch?v=Ea_Mkmj92PY
