# Erratum: Physical Literacy - A Journey of Individual Enrichment: An Ecological Dynamics Rationale for Enhancing Performance and Physical Activity in All

**DOI:** 10.3389/fpsyg.2020.633513

**Published:** 2020-12-11

**Authors:** Frontiers Production Office

**Affiliations:** Frontiers Media SA, Lausanne, Switzerland

**Keywords:** non-linear pedagogy, athletics skills model, motor learning, ecological psychology, executive function, physical education, sport coaching

Due to a production error, the reference “Woods et al., 2020” was incorrectly written as “Piaget, 1964” and “Woods et al., unpublished”. Both should be “Woods, C. T., Rudd, J., Robertson, S., and Davids, K. (2020). Wayfinding: how ecological perspectives of navigating dynamic environments can enrich our understanding of the learner and the learning process in sport. *Sports Med. Open* 6:51. doi: 10.1186/s40798-020-00280-9”.

The publisher apologizes for this mistake. The original article has been updated.
